# On the Status and Comparison of Glucose Intolerance in Female Breast Cancer Patients at Initial Diagnosis and during Chemotherapy through an Oral Glucose Tolerance Test

**DOI:** 10.1371/journal.pone.0093630

**Published:** 2014-04-01

**Authors:** Lin-jie Lu, Rui-jue Wang, Liang Ran, Lu Gan, Yang Bai, Liang-bin Jin, Zi-xiang Yao, Sheng-chun Liu, Guo-sheng Ren, Kai-nan Wu, Hong-yuan Li, Ling-quan Kong

**Affiliations:** The First Affiliated Hospital of Chongqing Medical University, Chongqing, China; University of Bari & Consorzio Mario Negri Sud, Italy

## Abstract

**Aims:**

This study is to estimate the status and comparison of glucose intolerance in female breast cancer patients at initial diagnosis and during chemotherapy through an oral glucose tolerance test (OGTT), as well as to learn the effect of chemotherapy on the glucose metabolism of breast cancer patients.

**Methods:**

All the 79 breast cancer patients at initial diagnosis, with the mean age of 53.2 years, and 96 breast cancer patients before the 5th or 6th cycle of chemotherapy, with the mean age of 51.5 years, participated in the study from December 2012 to October 2013. After an overnight fast, participants underwent OGTT test, and fasting and 2-hour glucose levels were measured to identify undiagnosed diabetes and prediabetes (i.e., impaired fasting glucose or impaired glucose tolerance) in them. Previously diagnosed diabetes among the female breast cancer patients was determined on the self-report and the medical record.

**Results:**

The overall incidences of total normal glucose tolerance, prediabetes, diabetes in female breast cancer patients at initial diagnosis and during chemotherapy were 24.1% and 38.5% (*p<0.05*), 50.6% and 28.1% (*p<0.05*), and 25.3% and 33.3% (*p>0.05*), respectively, and the differences of normal glucose tolerance and prediabetes instead of diabetes between the two groups were statistically significant. About 84% of the total diabetes and prediabetes in the female breast cancer patients at initial diagnosis and 79.7% of those during chemotherapy need to be diagnosed with OGTT.

**Conclusions:**

Breast cancer patients have high incidences of diabetes and prediabetes. After chemotherapy even with steroids, some breast cancer patients with abnormal glucose metabolism may even become normal. Isolated hyperglycemia 2 hours after glucose loading is common, and OGTT should be made for breast cancer patients at initial diagnosis and during chemotherapy.

## Introduction

Breast cancer is the commonest one of female malignancies worldwide and another major health problem in developed countries is diabetes mellitus, both with a raising tendency [1]–[3]. Diabetes is associated with multiple factors that may also be the risk factors of breast cancer, such as comorbidities, old age, lack of physical activity, obesity, low parity, genetic predisposition and metabolic syndrome [4]. Diabetes may take effects on breast cancer because of associated end organ damage which may influence therapy choices, affect therapeutic toxicities, and cause worse outcomes [5]–[7]. It was reported that about 16% of breast cancer patients worldwide had diabetes and that diabetic individuals tended to have poorer survival following treatment for breast cancer [8], [9]. Breast cancer patients often receive steroids as a component of their chemotherapy. It was believed that steroids and chemotherapeutics had profound effects on glucose metabolism, particularly on postprandial hyperglycemia [10], [11]. It was reported that, in the United States, impaired glucose tolerance was an independent predictor for cancer mortality [12] and even a few days of hyperglycemia had deleterious effects on the immune system [13].

In China, the statistical data indicated that the prevalences of total diabetes (both previously diagnosed diabetes and previously undiagnosed diabetes) and prediabetes in adults older than 20 years were 9.7% (10.6% among men and 8.8% among women) and 15.5% (16.1% among men and 14.9% among women), respectively. Epidemiologic studies suggest that type 2 diabetes (T2DM) increases breast cancer risk and goes along with an increased mortality [14]. The previously reported findings have not all been consistent, owing to methodological differences in sampling and to differences in the criteria used to define diabetes [15]. Furthermore, the prevalences of diabetes and prediabetes were probably under estimated in these studies because 2-hour oral glucose-tolerance tests (OGTT) were not performed in all participants [16]. It has been reported that isolated hyperglycemia 2 hours after glucose loading is common among Asian diabetic patients [17]. In the Shanghai of China diabetes study, 48.6% of patients with newly diagnosed diabetes had isolated hyperglycemia 2 hours after glucose loading, and 75.0% of those with prediabetes had isolated impaired glucose tolerance [18].

Our previous study on the status of total diabetes and prediabetes in breast cancer patients after systemic treatment through OGTT in Southwest of China showed that the overall incidences of total diabetes and prediabetes were 21.8% and 43.7%, respectively; about 80% of the diabetes were previously undiagnosed; about 80.0% of the cases of undiagnosed diabetes and prediabetes met the criteria for elevated 2-hour plasma glucose levels through OGTT instead of the criteria for elevated fasting glucose levels [16]. However, there have been no detailed studies about the status and comparison of isolated hyperglycemia 2 hours after glucose loading in breast cancer patients at initial diagnosis and during chemotherapy through OGTT.

This study is aimed to provide current and reliable data on the status of total diabetes (both previously diagnosed diabetes and previously undiagnosed diabetes) and prediabetes (i.e., impaired fasting glucose or impaired glucose tolerance) as well as the status of isolated hyperglycemia 2 hours after glucose loading in breast cancer patients at initial diagnosis and during chemotherapy through OGTT in China, and in the meantime, involves the research on the effect of chemotherapy on the glucose metabolism of breast cancer patients.

## Methods

### Study participants

This study were conducted in the Breast Cancer Center of Chongqing, the First Affiliated Hospital of Chongqing Medical University, where is situated in the southwest of China. There are approximately 31.4 million people who live in about 82,402.95 km^2^ area of Chongqing. This study was approved by The Ethics Committee of the First Affiliated Hospital of Chongqing Medical University, and all patients gave written informed consent. The female breast cancer patients received six cycles of TEC regimen (docetaxel, epirubicin and cyclophosphamide) or CEF regimen (cyclophosphamide, epirubicin and 5-flouracil) every three weeks. The women who were treated with docetaxel received 7.5 mg of dexamethasone (by oral administration) 24 hours, 12 hours, and, immediately before receiving docetaxel (per the product specification). All the 79 breast cancer patients at initial diagnosis, with the mean age of 53.2 years (ranging from 24 to 75), and 96 breast cancer patients, with the mean age of 51.5 years (ranging from 30 to 72), before the 5^th^ or 6^th^ cycle of chemotherapy, i.e., in about three weeks after the previous cycle of chemotherapy, participated in the study from December 2012 to October 2013. All subjects were informed about the purpose of the study and signed the informed consent form. After an overnight fast, all the breast cancer patients except the self-reported or medical-recorded patients with diabetes underwent OGTT, and fasting and 2-hour glucose levels were measured to identify undiagnosed diabetes and prediabetes (i.e., impaired fasting glucose or impaired glucose tolerance) in them. Previously diagnosed diabetes among the female breast cancer patients was determined on the self-report and the medical record.

### Oral glucose tolerance test

Participants were instructed to keep their usual physical activity and, diet for at least three days before OGTT. After at least 10 hours of overnight fasting, venous blood specimen was collected in a vacuum tube containing sodium fluoride, for the measurement of plasma glucose. Participants with no history of diabetes mellitus were given a standard 75-g glucose solution, blood samples were drawn at 0, 30, 60 and 120 minutes after the glucose load to measure glucose concentrations. Plasma glucose was measured at The Clinical Endocrine Laboratories of The First Affiliated Hospital of Chongqing Medical University. The laboratory successfully completed a standardization and certification program.

Their glucose intolerance was evaluated based on a 75 g OGTT according to the World Health Organization criteria: isolated impaired fasting glucose (IIFG, fasting glucose level, ≥6.1 mmol/L and <7.0 mmol/L, and 2-hour glucose level through OGTT <7.8 mmol/L), isolated impaired glucose tolerance (IIGT, fasting glucose level, <6.1 mmol/L, and, 2-hour glucose level through OGTT, ≥7.8 and <11.1 mmol/L), combined impaired fasting glucose and impaired glucose tolerance (CIFGIGT, fasting glucose level, ≥6.1 and <7.0 mmol/L, and 2-hour glucose level through OGTT, ≥7.8 and <11.1 mmol/L), and undiagnosed diabetes (fasting glucose level, ≥7.0 mmol/L, or 2-hour glucose level through OGTT, ≥11.1 mmol/L, or both). Total diabetes includes both previously diagnosed diabetes (PDD) and previously undiagnosed diabetes (PUD). Prediabetes was defined as either impaired fasting glucose (IFG) or, impaired glucose tolerance (IGT).

### Statistical Analysis

This study was designed to provide accurate estimations of the status of diabetes, prediabetes in the breast cancer patients at initial diagnosis and during chemotherapy in the southwest of China. The difference of the glucose metabolism status between female breast cancer patients at initial diagnosis and during chemotherapy were test by Chi-square test. SPSS 20.0 statistical software was used for analysis and a *P*<0.05 was considered significantly different.

## Results

**Figure 1:**
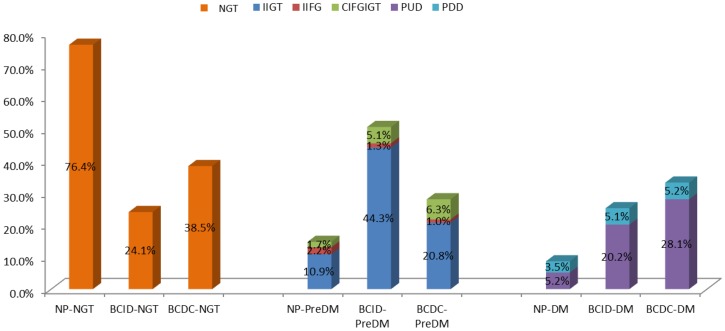
Status of glucose intolerance in female normal population and female adult breast cancer patients at initial diagnosis and during chemotherapy. Abbreviation: NP- Normal Population [14], BCID- breast cancer patients at initial diagnosis, BCDC- breast cancer patients during chemotherapy, NGT: Normal glucose tolerance, IIFG: Isolated impaired fasting glucose, IIGT: Isolated impaired glucose tolerance, CIFGIGT: combined impaired fasting glucose and impaired glucose tolerance, PUD: Previously undiagnosed diabetes, PDD: Previously diagnosed diabetes.

**Figure 2:**
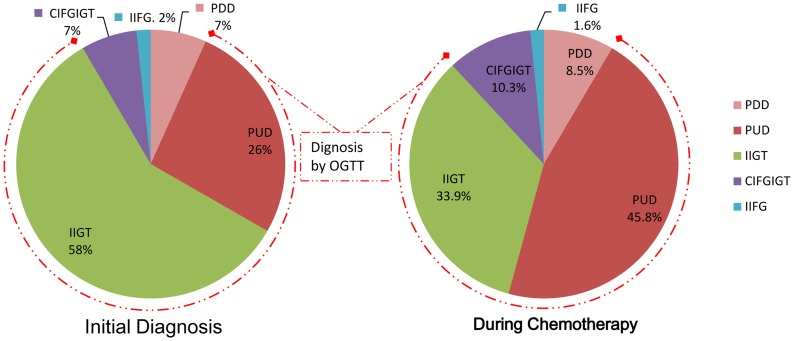
Component of diabetes and prediabetes among female breast cancer patents after initial diagnosis & during chemotherapy. Abbreviation: IIFG: Isolated impaired fasting glucose, IIGT: Isolated impaired glucose tolerance, CIFGIGT: Combined impaired fasting glucose and impaired glucose tolerance, PUD: Previously undiagnosed diabetes, PDD: Previously diagnosed diabetes.

The overall incidence of total diabetes (25.3%, including 5.1% PDD & 20.2% PUD) in 79 cases of breast cancer at initial diagnosis was lower than that (33.3%, including 5.2% PDD & 28.1% PUD) in 96 cases of breast cancer during chemotherapy, but without significant difference (*p>0.05*) (seen in Table 1). The overall incidences of total diabetes and prediabetes in the female breast cancer patients at initial diagnosis and during chemotherapy were 75.9% and 61.5%, respectively (seen in Fig.1). About 84.0% of the total diabetes and prediabetes in the female breast cancer patients at initial diagnosis and 79.7% of those during chemotherapy need only to be diagnosed with OGTT (seen in Fig.2). The incidences of previously undiagnosed diabetes in diabetic breast cancer patients at initial diagnosis and during chemotherapy were 80.0% and, 84.4%, respectively (seen in Fig.2). The incidence of prediabetes (50.6%) in breast cancer patients at initial diagnosis was significantly higher than that (28.1%) in those during chemotherapy (*p<0.05*), meanwhile, the incidences of IIGT in breast cancer patients with prediabetes at initial diagnosis and during chemotherapy were 87.5% and 74.1%,respectively (seen in Table 1 and Fig.2). The incidence of normal glucose tolerance (24.1%) in breast cancer patients at initial diagnosis was significantly lower than that (38.5%) in those during chemotherapy (*p<0.05*) (seen in Table 1).

**Table 1:** comparison of glucose intolerance in female breast cancer patients at initial diagnosis and during chemotherapy through OGTT.

	Normal glucose tolerance	Prediabetes	Diabetes	Total
Group of Initial diagnosis	19 (24.1%)*	40 (50.6%)*	20(25.3%)**	79 (100%)
Group of during chemotherapy	37 (38.5%)*	27 (28.1%)*	32(33.3%)**	96(100%)

*p<0.05,

** p>0.05.

The incidences of IIFG, IIGT, CIFGIGT, PUD and PDD, among these female adult breast cancer patients at initial diagnosis and during chemotherapy, were 1.3% & 1.0%, 44.3% & 20.8%, 5.1% & 6.3%, 20.2% & 28.1%, and 5.1% & 5.2%, respectively (seen in Fig.1).

## Discussion

Breast cancer is the commonest one of women malignancies worldwide and another major health problem in developed countries is diabetes, both with a raising tendency [1], [2]. Precise knowledge of the complex associations and interactions between diabetes and breast cancer, which may be named as breast oncodiabetology or, diabeto-oncology, is of great importance for their prevention and, treatment. Diabetes is clearly an overwhelming pandemic with deathly consequences. The risk of death among people with diabetes is twice that of people of a similar age without diabetes [19]. Diabetic adults have heart disease death rates that are 2 to 4 times higher than those of adults without diabetes, and the risk for stroke is also 2 to 4 times higher among people with diabetes [20]. T2DM doubles the risk of all-cause mortality and is also the leading cause of end-stage renal disease, blindness, and non-traumatic amputations [21]. It has been reported that diabetes has been associated with substantial premature death from several cancers, infectious diseases, and degenerative disorders [22]. In China, because of the rapid change in life style, there is concern that diabetes may become epidemic. The recent statistical data indicated that the prevalence of diabetes and prediabetes in female adults older than 20 years was 8.8% and 14.9%, respectively [23].

A link between diabetes and cancer was first proposed in 1934 and has been investigated extensively [1], [24], [25]. Epidemiologic studies suggest that diabetes increases breast cancer risk and goes along with an increased mortality [21]. It was reported that up to 16% of breast cancer patients worldwide had diabetes and that diabetic individuals tend to have poorer outcomes following treatment for breast cancer [8].

It is reported, in the United States, that compared with those having normal glucose tolerance, adults with impaired glucose tolerance had the greatest adjusted relative hazard of cancer mortality, suggesting that impaired glucose tolerance is an independent predictor for cancer mortality [12]. Some studies suggest that even a few days of hyperglycemia have deleterious effects on the immune system [13]. However, there has been limited studies about the accurate status and comparison of total diabetes (previously diagnosed diabetes plus undiagnosed diabetes), prediabetes, post-prandial hyperglycemia in breast cancer patients at initial diagnosis and during chemotherapy.

Our results indicate that diabetes has reached epidemic proportions in the female breast cancer patients at initial diagnosis and during chemotherapy in southwest of China. Of the 79 breast cancer patients at initial diagnosis and 96 cases during chemotherapy, the overall incidences of total diabetes and prediabetes in the female breast cancer patients at initial diagnosis and during chemotherapy were 75.9% and, 61.5%, respectively. The overall incidences of total diabetes (previously diagnosed plus previously undiagnosed diabetes) in the female breast cancer patients at initial diagnosis and during chemotherapy were 25.3% and 33.3%, respectively, which were obviously higher than that (8.7%) of normal Chinese population [23]. And in 80.0% of diabetic breast cancer patients at initial diagnosis and 84.4% of diabetic breast cancer patients during chemotherapy, the diabetes are previously undiagnosed. In addition, 50.6% of breast cancer patients at initial diagnosis and 28.1% of breast cancer patients during chemotherapy have prediabetes, which is an important risk factor for the development of overt diabetes and cardiovascular disease [26], [27]. While the prevalence of prediabetes in normal Chinese population was only 14.8% [23]. Randomized clinical trials have shown that interventions involving diet and exercise reduce the risk of diabetes among people with prediabetes [28], [29]. Public health measures should be undertaken to mitigate the consequences of new cases of diabetes in breast cancer patients at initial diagnosis and during chemotherapy. Importantly, about 75.9% of the female adult breast cancer patients at initial diagnosis and 61.5% of the female adult breast cancer patients during chemotherapy in china have diabetes and prediabetes, but only 5.1% of these patients at initial diagnosis and 5.2% of cases during chemotherapy were diagnosed as diabetes, with most of the diabetes and prediabetes undiagnosed. About 84% of the total diabetes and prediabetes in the female breast cancer patients at initial diagnosis and 79.7% of those during chemotherapy need only to be diagnosed with OGTT. So, a large multicentric study involving a whole country representative sample of Chinese breast cancer patients at initial diagnosis and during chemotherapy is needed, this may provide an accurate evaluation of the diabetes and prediabetes burden in Chinese female adult breast cancer patients at initial diagnosis and during chemothherapy.

During chemotherapy for breast cancer patients, the glucocorticoid dexamethasone is widely used to prevent side effects [30]. It is considered that steroids affect post-meal glucose much more so than morning fasting sugars and glucocorticoid administration is associated with impairment of insulin sensitivity, elevations in peripheral glucose levels, and the suppression of the hypothalamic – pituitary – adrenal axis [31]. It was reported that glucocorticosteroids (steroids) and chemotherapy had profound effects on glucose metabolism, particularly on postprandial hyperglycemia [10], [11]. Our results indicate that, the overall incidence of total diabetes (33.3%) in breast cancer patients during chemotherapy increased more obviously than that (25.3%) in breast cancer patients at initial diagnosis and the incidence of previously undiagnosed diabetes (84.4%) in diabetic breast cancer patients during chemotherapy were also much higher than that (80.0%) in breast cancer patients at initial diagnosis, but without statistical difference (*p>0.05*). Meanwhile, it is also found that the incidence of prediabetes (50.6%) in breast cancer patients at initial diagnosis was significantly higher than that (28.1%) in those during chemotherapy (*p<0.05*) and the incidence of normal glucose tolerance (24.1%) in breast cancer patients at initial diagnosis was significantly lower than that (38.5%) in those during chemotherapy (*p<0.05*). These findings suggest that though chemotherapeutics and steroids had certain effects on glucose metabolism, particularly on postprandial hyperglycemia, but without statistical difference, and most importantly, after chemotherapy even with steroids, some breast cancer patients with abnormal glucose metabolism may become normal with statistical difference. These may be related to the hypothesis of breast oncodiabetology that after chemotherapy and / or surgery, diabetes-inducing factors caused by breast cancer cells are completely or partially relieved and the reversible diabetes and prediabetes in breast cancer patients become alleviated or, even become normal, which need to be further studied.

These findings, which are firstly based on certain population based study involving one center representative sample of Chinese breast cancer women at initial diagnosis and during chemotherapy, should provide an firstly relative accurate estimation of the diabetes and, prediabetes burden in female adult breast cancer patients at initial diagnosis and during chemotherapy in southwest of China. In addition, the diagnosis of diabetes and prediabetes were firstly established on the basis of both fasting plasma glucose levels and 2-hour plasma glucose levels in an oral glucose-tolerance test, and these measurements were obtained with the use of stringent quality control procedures.

Several previous studies have documented high prevalence of diabetes in breast cancer patients [32]. However, in those studies, OGTT were not performed in the entire study population; therefore, the true incidence of undiagnosed diabetes may have been underestimated. Therefore, in the previous research, we firstly studied the status of total diabetes and prediabetes in breast cancer patients after systemic treatment through OGTT in Southwest of China and found that the overall incidences of total diabetes and prediabetes were 21.8% and, 43.7%, respectively; about 80% of the diabetes were previously undiagnosed; about 80.0% of the cases of undiagnosed diabetes and, prediabetes met the criteria for elevated 2-hour plasma glucose levels through OGTT but not the criteria for elevated fasting glucose levels [16].

In the present study, 80.0% of the cases of diabetes and, 87.5% of the cases of prediabetes in breast cancer patients at initial diagnosis as well as 84.4% of the cases of diabetes and 74.1% of the cases of prediabetes in breast cancer patients during chemotherapy met the criteria for elevated 2-hour plasma glucose levels through OGTT, but not the criteria for elevated fasting glucose levels. Even accounting for differences in diagnostic criteria, our study firstly documents high incidences of previously undiagnosed diabetes and prediabetes in female adult breast cancer patients at initial diagnosis and during chemotherapy in Southwest of China.

It has been suggested that isolated hyperglycemia 2 hours after glucose loading is common among Asian patients with diabetes. It was found that in a pooled analysis of Asian cohorts, more than half of the subjects with diabetes had isolated hyperglycemia 2 hours after glucose loading and three quarters of the subjects with prediabetes had isolated impaired glucose tolerance [17].The latest statistical data for Chinese population also indicated that 46.6% of the cases of diabetes and 70.7% of the cases of prediabetes met the criteria for elevated 2-hour plasma glucose levels in OGTT but not the criteria for elevated fasting glucose levels, and documented a large increase in the prevalences of previously diagnosed diabetes, previously undiagnosed diabetes, and prediabetes in Chinese population [23]. Our results firstly indicate that about 80% of the female adult diabetic breast cancer patients at initial diagnosis and 84.4% of the female adult diabetic breast cancer patients during chemotherapy had isolated hyperglycemia 2 hours after glucose loading as well as 87.5% of the female adult breast cancer patients with prediabetes at initial diagnosis and 74.1% of the subjects with prediabetes during chemotherapy had isolated impaired glucose tolerance, which are much higher than those of Asian and Chinese normal population. This means that without OGTT, most of the diabetes and prediabetes in adult breast cancer women at initial diagnosis and during chemotherapy cannot be confirmed, therefore, OGTT should be made for the breast cancer patients at initial diagnosis and during chemotherapy (seen in Fig.2).

This study has several limitations. First, it is only small samples and one center based prospective study on the incidences of diabetes and prediabetes in women breast cancer patients at initial diagnosis and during chemotherapy, and a large samples and multicenter-based study involving a nationally representative samples of Chinese female breast cancer patients are needed. Second, comparison of glucose intolerance in female breast cancer patients at initial diagnosis and during chemotherapy through OGTT was not carried out in the same patient.

In summary, our results show that diabetes and prediabetes are highly prevalent in the female adult breast cancer patients at initial diagnosis and during chemotherapy in southwest of China. More troublesome is the finding that the majority of cases of diabetes and prediabetes in the breast cancer women at initial diagnosis and during chemotherapy in southwest of China are undiagnosed. These results indicate that diabetes has become a major public health problem in breast cancer patients and that strategies aimed at the prevention, detection, and treatment of diabetes in breast cancer patients at initial diagnosis and during chemotherapy are needed in China. Chemotherapeutics and steroids had certain effects on glucose metabolism, particularly on postprandial hyperglycemia, but most importantly, after chemotherapy even with steroids, some breast cancer patients with abnormal glucose metabolism may become normal. Isolated hyperglycemia 2 hours after glucose loading is common, and, OGTT should be made for the breast cancer patients at initial diagnosis and during chemotherapy.

## References

[1] A M (1934) Diabetes and cancer. New Engl J Med 211: 339–349.

[2] Schott S, Schneeweiss A, Sohn C (2010) Breast cancer and diabetes mellitus. Exp Clin Endocrinol Diabetes 118: 673–677. 20533174 10.1055/s-0030-1254116

[3] Ferlay J, Shin HR, Bray F, Forman D, Mathers C, et al (2010) Estimates of worldwide burden of cancer in 2008: GLOBOCAN 2008. International journal of cancer 127: 2893–2917. 21351269 10.1002/ijc.25516

[4] Ahn J, Schatzkin A, Lacey JV Jr, Albanes D, Ballard-Barbash R, et al (2007) Adiposity, adult weight change, and postmenopausal breast cancer risk. Arch Intern Med 167: 2091–2102. 17954804 10.1001/archinte.167.19.2091

[5] Lipscombe LL, Goodwin PJ, Zinman B, McLaughlin JR, Hux JE (2008) The impact of diabetes on survival following breast cancer. Breast Cancer Res Treat 109: 389–395. 17659440 10.1007/s10549-007-9654-0

[6] Erickson K, Patterson RE, Flatt SW, Natarajan L, Parker BA, et al (2011) Clinically defined type 2 diabetes mellitus and prognosis in early-stage breast cancer. J Clin Oncol 29: 54–60. 21115861 10.1200/JCO.2010.29.3183 PMC3055860

[7] Schrauder MG, Fasching PA, Haberle L, Lux MP, Rauh C, et al (2011) Diabetes and prognosis in a breast cancer cohort. J Cancer Res Clin Oncol 137: 975–983. 21132511 10.1007/s00432-010-0960-2 PMC11827943

[8] Wolf I, Sadetzki S, Catane R, Karasik A, Kaufman B (2005) Diabetes mellitus and breast cancer. Lancet Oncol 6: 103–111. 15683819 10.1016/S1470-2045(05)01736-5

[9] Yerrabothala S, Shaaban H, Capo G, Maroules M, Debari VA (2013) The Impact of Diabetes Mellitus on Breast Cancer Outcomes: A Single Center Retrospective Study. Pathol Oncol Res. 10.1007/s12253-013-9666-5 23832821

[10] Hickish T, Astras G, Thomas P, Penfold S, Purandare L, et al (2009) Glucose intolerance during adjuvant chemotherapy for breast cancer. J Natl Cancer Inst 101: 537. 19318627 10.1093/jnci/djp025

[11] Oyer DS, Shah A, Bettenhausen S (2006) How to manage steroid diabetes in the patient with cancer. J Support Oncol 4: 479–483. 17080737

[12] Saydah SH, Loria CM, Eberhardt MS, Brancati FL (2003) Abnormal glucose tolerance and the risk of cancer death in the United States. Am J Epidemiol 157: 1092–1100. 12796045 10.1093/aje/kwg100

[13] Furnary AP, Wu Y, Bookin SO (2004) Effect of hyperglycemia and continuous intravenous insulin infusions on outcomes of cardiac surgical procedures: the Portland Diabetic Project. Endocr Pract 10 Suppl 221–33. 15251637 10.4158/EP.10.S2.21

[14] Yang W, Lu J, Weng J, Jia W, Ji L, et al (2010) Prevalence of diabetes among men and women in China. New England Journal of Medicine 362: 1090–1101. 20335585 10.1056/NEJMoa0908292

[15] Arif JM, Al-Saif AM, Al-Karrawi MA, Al-Sagair OA (2011) Causative relationship between diabetes mellitus and breast cancer in various regions of Saudi Arabia: an overview. Asian Pac J Cancer Prev 12: 589–592. 21627349

[16] Ji GY, Jin LB, Wang RJ, Bai Y, Yao ZX, et al (2013) Incidences of diabetes and prediabetes among female adult breast cancer patients after systemic treatment. Med Oncol 30: 687. 23925668 10.1007/s12032-013-0687-4

[17] Qiao Q, Nakagami T, Tuomilehto J, Borch-Johnsen K, Balkau B, et al (2000) Comparison of the fasting and the 2-h glucose criteria for diabetes in different Asian cohorts. Diabetologia 43: 1470–1475. 11151755 10.1007/s001250051557

[18] Jia WP, Pang C, Chen L, Bao YQ, Lu JX, et al (2007) Epidemiological characteristics of diabetes mellitus and impaired glucose regulation in a Chinese adult population: the Shanghai Diabetes Studies, a cross-sectional 3-year follow-up study in Shanghai urban communities. Diabetologia 50: 286–292. 17180353 10.1007/s00125-006-0503-1

[19] Lozano R, Naghavi M, Foreman K, Lim S, Shibuya K, et al (2012) Global and regional mortality from 235 causes of death for 20 age groups in 1990 and 2010: a systematic analysis for the Global Burden of Disease Study 2010. Lancet 380: 2095–2128. 23245604 10.1016/S0140-6736(12)61728-0 PMC10790329

[20] Norhammar A, Schenck-Gustafsson K (2013) Type 2 diabetes and cardiovascular disease in women. Diabetologia 56: 1–9. 10.1007/s00125-012-2694-y 22945305

[21] Srokowski TP, Fang S, Hortobagyi GN, Giordano SH (2009) Impact of diabetes mellitus on complications and outcomes of adjuvant chemotherapy in older patients with breast cancer. J Clin Oncol 27: 2170–2176. 19307509 10.1200/JCO.2008.17.5935 PMC2674004

[22] Emerging Risk Factors C, Seshasai SR, Kaptoge S, Thompson A, Di Angelantonio E, et al (2011) Diabetes mellitus, fasting glucose, and risk of cause-specific death. N Engl J Med 364: 829–841. 21366474 10.1056/NEJMoa1008862 PMC4109980

[23] Yang SH, Dou KF, Song WJ (2010) Prevalence of diabetes among men and women in China. N Engl J Med 362: : 2425–2426; author reply 2426. 20578276

[24] Malek M, Aghili R, Emami Z, Khamseh ME (2013) Risk of Cancer in Diabetes: The Effect of Metformin. ISRN Endocrinol 2013: 636927. 24224094 10.1155/2013/636927 PMC3800579

[25] Buysschaert M, Sadikot S (2013) Diabetes and cancer: A 2013 synopsis. Diabetes Metab Syndr 7: 247–250. 24290094 10.1016/j.dsx.2013.08.001

[26] Schmidt MI, Duncan BB, Bang H, Pankow JS, Ballantyne CM, et al (2005) Identifying individuals at high risk for diabetes: The Atherosclerosis Risk in Communities study. Diabetes Care 28: 2013–2018. 16043747 10.2337/diacare.28.8.2013

[27] Levitzky YS, Pencina MJ, D'Agostino RB, Meigs JB, Murabito JM, et al (2008) Impact of impaired fasting glucose on cardiovascular disease: the Framingham Heart Study. J Am Coll Cardiol 51: 264–270. 18206734 10.1016/j.jacc.2007.09.038

[28] Tuomilehto J, Lindstrom J, Eriksson JG, Valle TT, Hamalainen H, et al (2001) Prevention of type 2 diabetes mellitus by changes in lifestyle among subjects with impaired glucose tolerance. N Engl J Med 344: 1343–1350. 11333990 10.1056/NEJM200105033441801

[29] Li G, Zhang P, Wang J, Gregg EW, Yang W, et al (2008) The long-term effect of lifestyle interventions to prevent diabetes in the China Da Qing Diabetes Prevention Study: a 20-year follow-up study. Lancet 371: 1783–1789. 18502303 10.1016/S0140-6736(08)60766-7

[30] Ioannidis JP, Hesketh PJ, Lau J (2000) Contribution of dexamethasone to control of chemotherapy-induced nausea and vomiting: a meta-analysis of randomized evidence. J Clin Oncol 18: 3409–3422. 11013282 10.1200/JCO.2000.18.19.3409

[31] Munck A (1971) Glucocorticoid inhibition of glucose uptake by peripheral tissues: old and new evidence, molecular mechanisms, and physiological significance. Perspect Biol Med 14: 265–269. 5546253 10.1353/pbm.1971.0002

[32] Zhang PH, Chen ZW, Lv D, Xu YY, Gu WL, et al (2012) Increased risk of cancer in patients with type 2 diabetes mellitus: a retrospective cohort study in China. BMC Public Health 12: 567. 22839452 10.1186/1471-2458-12-567 PMC3487805

